# Scales Used to Measure Job Stressors in Intensive Care Units: Are They Relevant and Reliable? A Systematic Review

**DOI:** 10.3389/fpsyg.2020.00245

**Published:** 2020-03-12

**Authors:** Alexandra Laurent, Florent Lheureux, Magali Genet, Maria Cruz Martin Delgado, Maria G. Bocci, Alessia Prestifilippo, Guillaume Besch, Gilles Capellier

**Affiliations:** ^1^Le Laboratoire de Psychologie: Dynamiques Relationnelles Et Processus Identitaires (Psy DREPI), University of Bourgogne Franche-Comté, Dijon, France; ^2^La Maison des Sciences de l'Homme et de l'Environnement (MSHE) C. N. Ledoux, University of Bourgogne Franche-Comté, Besançon, France; ^3^Laboratory of Psychology, University of Bourgogne Franche-Comté, Besançon, France; ^4^Intensive Care Unit, Hospital Universitario de Torrejón, Madrid, Spain; ^5^Department of Anesthesiology and Intensive Care, Fondazione Policlinico Universitario Agostino Gemelli IRCCS, Rome, Italy; ^6^Fondazione Policlinico Universitario Agostino Gemelli IRCCS, Rome, Italy; ^7^Department of Anesthesiology and Intensive Care Medicine, University Hospital of Besançon, University of Bourgogne Franche-Comté, Besançon, France; ^8^Medical Intensive Care Unit, University Hospital of Besançon, Besançon, France

**Keywords:** systematic review, intensive care unit (ICU), job stressors, occupational stressors, psychometrics, job stress scales

## Abstract

**Background:** Many studies have been conducted in intensive care units (ICUs) to identify the stress factors involved in the health of professionals and the quality and safety of care. The objectives are to identify the psychometric scales used in these studies to measure stressors and to assess their relevance and validity/reliability.

**Methods:** All peer-reviewed full-text articles published in English between 1997 and 2016 and focusing on an empirical quantitative study of job stressors were identified through searches on seven databases and editorial portals.

**Results:** From the 102 studies analyzed, we identified 59 different scales: 17 “all settings scales” (16 validated scales), 20 “healthcare settings scales” (13 validated scales), and 22 “ICU settings scales” (two validated scales). All these scales used measured stressors from at least one of the following eight broad categories: High job demands, Problematic relationships with other professionals, Lack of control over work situations and career, Lack of organizational resources, Problematic situations with users and relatives, Dealing with ethical- and moral-related situations, Risk management issues, and Disadvantages in comparison to other occupational situations. The “all settings scales” and “healthcare settings scales,” the most often validated, did not measure, or only slightly measured, the stressors most specific to ICUs. Where these were taken into account, the authors were forced to develop their own tools or modify existing scales without testing the validity of the tool used.

**Conclusions:** This review highlights the lack of a tool that meets both the criteria of validity and relevance with regard to the specificity of work in ICUs. Future research must focus on developing reliable/valid tools covering all types of relevant stressors to ensure the quality of the studies carried out in this field.

## Introduction

Intensive care unit (ICU) professionals must deal with patients with serious medical conditions that require complex diagnostic and therapeutic procedures. This often requires a considerable level of coordination of human resources. Furthermore, end-of-life decisions are frequent and contribute to an intense emotional charge (Teixeira et al., 2014; Flannery et al., 2016). The ICU is thus fertile ground for the emergence of professional stressors (Donchin and Seagull, 2002; Embriaco et al., 2007a). Assessed by individuals as situations that weaken or are beyond their resources (Lazarus and Folkman, 1984), work-related stressors impact the mental and physical health of workers and the quality and safety of care (Sochalski, 2004; de Cássia Pereira Fernandes et al., 2016; Krämer et al., 2016; Dragano et al., 2017; Vandevala et al., 2017).

To assess these professional stressors, various surveys have been developed that cover either generic scales addressing all professional activities or more specific scales focusing primarily on the healthcare field or on a specific healthcare sector. These healthcare scales are used to measure specific stressors such as “end-of-life decisions” (Ozden et al., 2013; Teixeira et al., 2014), “conflicts,” or the “health culture” (Profit et al., 2014; Garrouste-Orgeas et al., 2015) particularly studied for their involvement in burnout or anxiety–depression.

While the identification of professional stressors is in line with the promotion of well-being at work and the quality of healthcare, the reliability of the psychometric tools is also of great importance. However, it appears that the multiplication of stress assessment tools makes it difficult to choose the most appropriate scale. In this sense, Bonneterre et al. (2008) denounce the use of unsuitable tools or the use of tools with insufficient psychometric qualities, which make the predictive validities between stressors and epidemiological indicators unreliable. Therefore, when researchers or clinicians wish to evaluate the stressors present in the ICU, what types of tools can they find in the literature? First, are these tools psychometrically valid? In addition, are they able to measure all relevant stressors, including those most specific to ICUs? This latter question deals with the issue of ecological validity. The ecological validity concept examined whether a study and its findings are representative of real-life situations (Brewer and Crano, 2014). In particular, ecologically valid studies used material and procedure that satisfy three parameters (Schmuckler, 2001): they reflect situations or events that can actually occur in participants' everyday lives, reproduce or they refer to features of participants' current living environments, and stimulate from participants reactions that are already available in their response repertoire. Applied to the measurement of stressors in ICUs, an ecologically valid tool should cover all types of situations actually occurring in ICUs that are likely to cause stress to professionals, with item content that refers to their current working environment (e.g., service functioning, patients and families, tasks to perform). Thus, the ideal tool would respect these principles in addition to present good psychometric properties (e.g., reliability, construct validity). But is it possible to find such an ideal tool in peer-reviewed journals?

To answer these questions, our literature review was guided by the following research objectives:

1. To comprehensively identify the scales and questionnaires that have been used to date to measure perceived job stressors in the ICU (Are there few or numerous measurement tools in the peer-reviewed literature?).
2. To determine the most frequently and least frequently used scales/questionnaires (Is there a consensual use of one or several tools? If so, what may explain this?).
3. To critically examine the ecological validity of these studies, i.e., their ability to take into account all stressors relevant to ICU settings; this means specific stressors as well as more general stressors such as job demands, lack of social support, etc.
4. To critically examine their basic psychometric reliability/validity as evidenced by the use of suitable methodological and statistical procedures such as factor analysis, Cronbach's alpha, etc.

## Methods

### Study Design

We considered all peer-reviewed full-text articles reporting an empirical study, a literature review, or a meta-analysis published in English between 1997 and 2016. The choice of article in English corresponds to the objective of reporting on the most commonly used stress scales in intensive care at an international level. In this sense, English publications, the common language of international researchers and practitioners, include the largest number of journals with high international visibility. Only the empirical studies that used a quantitative methodology to explicitly measure job/occupational stress factors for healthcare professionals in ICUs were selected.

Noncompliant articles (commentaries, case reports, posters, and editorials) simply reporting perceived stress or focusing on patients instead of healthcare providers were excluded.

### Search Strategy and Study Eligibility

The search for and selection of articles were conducted between March and May 2017 (according to PRISMA guidelines). Seven databases and editorial portals (Medline via Pubmed; PsycInfo and Psychology, and behavioral science collection via EBSCOhost; Elsevier's ScienceDirect, SpringerLink, Sage Journals, and Wiley Online Library) were screened using the following terms in the title, the abstract, or the keywords: (intensive OR critical) AND care AND (professional OR job OR work OR occupational) AND (stress OR stressors OR burnout). The references of 10 literature review articles (Dunser et al., 2006; Embriaco et al., 2007b; Fassier and Azoulay, 2010; Adriaenssens et al., 2015; Karanikola et al., 2015; Van Mol et al., 2015; Chuang et al., 2016; Flannery et al., 2016) were manually screened to identify other relevant studies that had not been initially retrieved.

**Figure 1:**
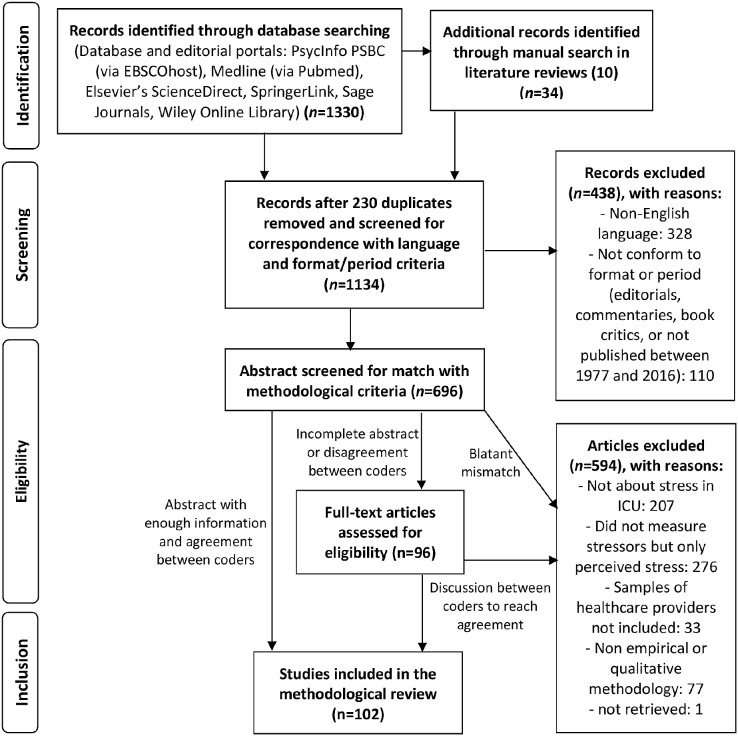
Flow diagram of selected articles based on relevance search criteria.

The initial search, conducted by MG, identified 1,330 records (Figure 1), and 34 were added following a manual search in the references of the articles retrieved. Duplicates (*n* = 230), articles in a language other than English (*n* = 328), or those not conforming to the search criteria (*n* = 110) were removed. The abstracts were then screened for eligibility separately by two authors (MG and FL). When disagreement or the need for further analysis arose, the entire article was (*n* = 96) examined and discussions were held between AL, FL, MG, MM, MB, and AP to reach an agreement. From the 696 articles thus selected, 594 were excluded as they did not focus on stress in the ICU (*n* = 207), did not measure stressors (*n* = 276), did not include healthcare providers (*n* = 33), did not use a quantitative methodology (*n* = 77), or could not be retrieved (*n* = 1). In total, our review focused on 102 empirical studies (references in Supplementary Material).

### Data Extraction and Criteria Used to Assess the Quality and the Relevance of Existing Scales in Intensive Care Unit Contexts

Following the selection of the 102 relevant articles, data were extracted and coded by AL and FL for each scale according to five criteria:

- *Identification of the scale used* and the *number of articles* that used it.
- The *origin and metrological reliability/validity*. At that level, we made a distinction between *ante hoc* and *ad hoc* scales, with subdivisions within these two broad categories of scales. This is because “previously-validated scales are generally preferable to *ad hoc* scales” (Furr, 2011, p. 8) given that the quality assessment of *ad hoc* scales rarely goes beyond face validity as perceived by the researchers (i.e., lack of independent evidence of validity from other sources such as experts or participants). Furthermore, the use of *ad hoc* measures reduces the comparability of studies as it introduces a possible confounding factor. Moreover, they generally lack a complete inspection of other forms of validity and reliability, as estimated by internal consistency coefficients (e.g., Cronbach's alpha), high test–retest correlation coefficients, theory-consistent and interpretable factor structure without problematic loadings, or correlations with similar measures (convergent validity) or logically related phenomena (nomological validity). In this respect, “rigorously developed measures have a lower probability of being based on chance or method variance than *ad hoc* measure” Peter and Churchill (1986, p. 3). Finally, *ante hoc* scales offer more the guarantee that they were elaborated independently from any hypothesis-testing purpose (i.e., absence of hypothesis confirmation bias in the generation of items). In addition, we also consider whether *ante hoc* validated scales were modified—or not—by ICU researchers. In fact, as pointed out by Furr (2011, p. 9) “well-validated original scales are preferable to modified scales. Because a modified scale's psychometric properties and quality might differ from those of the original scale, the modified scale is—to some degree—an *ad hoc* scale. As such, its psychometric properties and quality are unclear and suspect.” Nevertheless, because some authors using an *ad hoc* scale have provided validity-related information regarding the origin of items and/or basic statistics (generally Cronbach's alpha and sometimes factor analysis results), we differentiated *ad hoc* scales according to the presence or absence of such information. As a result of the combination of these criteria, we differentiated five types of scales ranging from the probably more reliable/valid to the less reliable/valid: *ante hoc* validated scales (Type 1); *ante hoc* validated scales with *ad hoc* modifications (items removed or rewritten) (Type 2); *ad hoc* scales with validity-related information regarding the origin of items and basic statistics (Type 3); *ad hoc* scales with validity-related information regarding the origin of items only *or* report of basic statistics only (Type 4); *ad hoc* scales without report of any validity-related information (Type 5).
- The *target population* and the *level of generality or specificity* according to three levels: (1) scales designed to measure stressors in all work settings (i.e., all settings scales); (2) scales specific to healthcare providers from several types of clinical units/settings (i.e., healthcare settings scales); (3) scales tailored to measure stressors specific to ICU settings.
- The *number of items* and, when relevant and available, their subscales.
- The *types of stressors* measured. An in-depth assessment of the subscales and items enabled us to develop a typology of all stressors that had been measured by the different scales. FL, AL, and MG examined in detail each scale with regard to its structure (presence and labels of subscales) and item content and subsequently made iterative comparisons between them to identify the broad types and subtypes of stressors measured when considering all scales together. The choice of an inductive methodology to define the typology of stressors made it possible to allow oneself not to be limited to a typology preconceived by a theoretical model, which would risk not covering all the stressors explored in the literature. This typology was then critically examined by AL, GC, and GB and revised subsequently to reach a consensus. Then, each scale was assessed using a binary format “yes/no presence” for each type and subtype of stressors. This criterion was especially considered to assess the ecological validity of scales. Because ICU professionals can be jointly exposed to widely common stressors (e.g., workload, lack of support from colleagues), stressors shared with many other health professions (e.g., lack of recognition from patients and families, administrative hassles), and stressors more typical of ICUs (e.g., end-of-life decisions, constant monitoring of critically ill patients), we consider that the more a scale covers exhaustively the different types and subtypes of stressors, the more it was considered as ecologically valid.

## Results

### What Types of Scales Are Used in the Intensive Care Unit?

From the 102 studies analyzed in our literature review, we identified 59 different scales according to three main categories (Table 1): 17 “generic scales” used in all sectors of professional activities, 20 “healthcare scales” used in the field of human health, and 22 scales specific to ICU settings. Only 28 out of the 59 scales were Type 1 scales (*ante hoc* validated). The majority of them were generic scales (13/17) or healthcare settings scales (13/20); there were only two ICU-specific scales (2/22).

**Table 1:** Scale description according to assessment criteria.

**Scale**	**Number of studies**	**References in online Supplementary Material**	**Metrological validity**	**Type of setting**	**Type of population**	**Stressors defined and measured with numbers of items**
Job Content Questionnaire (JCQ) and variants	17 of which 5 used with another scale and 3 not used entirely (12-item version)	(3, 5–7, 17, 18, 30, 36, 40, 41, 51, 61, 64, 71, 76, 87, 92)	Type 1 (14 articles) Type 2 (3 articles)	All settings	All professionals	12, 29, or 49 items with three to five subscales: Psychological and physical demands of the job, decision latitude, social support, job insecurity. *Types of stressors measured*: Lack of positive/supportive relationship with colleagues, Lack of instrumental support from supervisor, Conflict with colleagues, Conflict with supervisor, Workload/time pressure, Interruption/interference/distraction/unanticipated changes and resequencing, Task complexity/High level of attention/performance, Lack of decision authority/autonomy (timing, method, etc.), Lack of growth opportunities, Skill underutilization, Lack of task diversity/interest, Lack of task–role clarity, Lack of job security
Effort–Reward Imbalance (ERI) questionnaire	3	(39, 51, 95)	Type 1	All settings	All professionals	23 items with 3 subscales: Extrinsic effort, extrinsic reward, and overcommitment. *Types of stressors measured*: Lack of instrumental support from colleagues, Lack of positive/supportive relationship with supervisor, Workload/time pressure, Interruption/interference/distraction/unanticipated changes and resequencing, High managerial/decisional responsibilities, Lack of growth opportunities, Skill underutilization, Lack of job security
Workplace Stress Scale (WSS)	2	(1, 79)	Type 1	All settings	All professionals	12 items with 2 subscales: Job demands and Job Resources. *Types of stressors measured*: Workload/time pressure, Role conflicts/contradictory demands, Lack of decision authority/autonomy (timing, method, etc.), Risky situations for oneself, Lack of job security
Copenhagen Psychosocial Questionnaire V2 (COPSOQ II)	2	(13, 69)	Type 1	All settings	All professionals	128 items with 3 subscales: work demands, health, job outcomes. *Types of stressors measured*: Lack of instrumental support from colleagues, Lack of positive/supportive relationship with supervisor, Lack of team cohesion, Lack of instrumental support from supervisor, Conflict with colleagues, Death and suffering and emotion regulation, Communicating with and fulfilling the emotional needs of users or relatives, Conflict with supervisor, Injustice, discrimination, harassment, bullying, Workload/time pressure, Task complexity/High level of attention/performance, Role conflicts/contradictory demands, Lack of participation in workplace and service-level policies, Lack of decision authority/autonomy (timing, method, etc.), Lack of growth opportunities, Lack of task diversity/interest, Lack of a predictable, stable and recovery-propitious schedule, Lack of predictable and stable work relationships and place, lack of task–role clarity, Lack of task meaning/utility lack of hierarchical role clarity, Observing deviations from safety standards, Lack of job security, Work–Home conflict (e.g., because of night shifts, on site call)
Daily Hassles Questionnaire (DHQ)	1	(100)	Type 1	All settings	All professionals	118 items with no identified subscales. *Types of stressors measured*: Lack of positive/supportive relationship with colleagues, Conflict with supervisor, Injustice, discrimination, harassment, bullying, Workload/time pressure, Interruption/interference/distraction/unanticipated changes and resequencing, Task complexity/High level of attention/performance, Skill underutilization, Lack of job security, Work–Home conflict (e.g., because of night shifts, onsite call)
NASA Task Load Index (NASA-TLX)	1 used with another scale	(63)	Type 1	All settings	All professionals	6 items with 6 subscales: mental demands, physical demands, temporal demands, performance, effort, and frustration. *Types of stressors measured*: Workload/time pressure, Task complexity/High level of attention/performance, Physical efforts during task performance
Occupational Stress Indicator (OSI)	1	(29)	Type 1	All settings	All professionals	167 items with 7 subscales: sources of pressure, Type A behavior, locus of control, coping strategies, job satisfaction, mental health, and physical health. *Types of stressors measured*: Lack of positive/supportive relationship with colleagues, Lack of instrumental support from colleagues, Conflict with colleagues, Conflict with inappropriate expectations or behaviors from users (customer, client, patient, etc.), Workload/time pressure, Taxing work environment (noisy, hectic, crowded, heated, etc.), Role conflicts/contradictory demands, High managerial/decisional responsibilities, Lack of growth opportunities, Lack of task-related skills or preparation (incl. training, knowledge update), Skill underutilization, Lack of a predictable, stable, and recovery-propitious schedule, lack of task–role clarity, Risky situations for oneself, Lack of pride/self-respect
Brief job stress questionnaire (B-JSQ)	1	(43)	Type 1	All settings	All professionals	84 items with 4 subscales: job-related stress factors and social support, psychological and somatic symptom. *Types of stressors measured*: Lack of team cohesion, Lack of or inappropriate inter-services/administrative collaboration, Workload/time pressure, Task complexity/High level of attention/performance, Taxing work environment (noisy, hectic, crowded, heated, etc.), Physical efforts during task performance, Lack of participation in workplace and service-level policies, Lack of decision authority/autonomy (timing, method, etc.), Skill underutilization
Rizzo et al. (1970) Role ambiguity and role conflict scales	1 used with another scale	(67)	Type 1	All settings	All professionals	14 items with 2 subscales: role ambiguity and role conflict. *Types of stressors measured*: Lack of team cohesion, Role conflicts/contradictory demands, Lack of staffing, Lack of information (e.g., to ask patients' questions), Lack of or low-quality or low accessibility to material resources, lack of task–role clarity, Lack of adequate rules and procedures, Lack of task meaning/utility, lack of hierarchical role clarity
Instrument for STress-oriented Analysis (ISTA)	1	(46)	Type 1	All settings	All professionals	30 items with 2 subscales: stressors and resources. *Types of stressors measured*: Lack of positive/supportive relationship with colleagues, Lack of instrumental support from colleagues, Task complexity/High level of attention/performance, Taxing work environment (noisy, hectic, crowded, heated, etc.), Role conflicts/contradictory demands, Lack of decision authority/autonomy (timing, method, etc.), Lack of task diversity/interest, Lack of or low-quality or low accessibility to material resources, Risk of making severe errors, Risky situations for oneself
Job Control Scale (JCS)	1 used with another scale and not used entirely	(93)	Type 1	All settings	All professionals	10 items with 2 subscales: timing control and method control. *Type of stressors measured*: Lack of decision authority/autonomy (timing, method, etc.)
Negative Acts Questionnaire-Revised (NAQ-R)	1	(24)	Type 1	All settings	All professionals	22 items with subscales about work-related bullying, person-related bullying, or physical intimidation, respectively. *Types of stressors measured*: Conflict with colleagues, Conflict with supervisor, Injustice, discrimination, harassment, bullying, Workload/time pressure, Lack of participation in workplace and service-level policies, Skill underutilization, Lack of information (e.g., to ask patients' questions)
Interpersonal Work Relations Scale (in Portuguese ERIT)	1 used with another scale	(70)	Type 1	All settings	All professionals	17 items with 2 subscales: sociability and feeling about oneself. *Types of stressors measured*: Lack of positive/supportive relationship with colleagues, Lack of instrumental support from colleagues
Supervisor version of the Survey Perceived Organizational Support (SPOS)	1	(93)	Type 1	All settings	All professionals	4 items (partial) with no subscale. *Type of stressors measured*: Lack of positive/supportive relationship with supervisor
NIPG Questionnaire for Work Content–Well-being at Work (in Dutch NOVA-WEBA)	1 not used entirely	(90)	Type 2	All settings	All professionals	7 items (partial) with 2 subscales: autonomy and work pressure. *Types of stressors measured*: Workload/time pressure, Lack of decision authority/autonomy (timing, method, etc.)
Work Experience and Assessment Questionnaire (in Dutch VBBA)	1 not used entirely	(90)	Type 2	All settings	All professionals	15 items (partial) with 4 subscales: emotional demands, physical demands, social support, development opportunities. *Types of stressors measured*: Lack of growth opportunities, Physical efforts during tasks performance, Death and suffering and emotion regulation, Communicating with and fulfilling the emotional needs of users or relatives
Perceived Job Stressors (PJS)–Negative Subscale	1 used with another scale and not used entirely	(78)	Type 2	All settings	All professionals	9 items with no identified subscales. *Types of stressors measured*: Workload/time pressure, Interruption/interference/distraction/unanticipated changes and resequencing, Role conflicts/contradictory demands, High managerial/decisional responsibilities, Underload
Moral Distress Scale Revised (MDS-R)	15 of which 3 used with another scale	(11, 15, 16, 19, 20, 33, 42, 57, 60, 62, 68, 82, 86, 98, 99)	Type 1	Healthcare settings	Healthcare professionals	18 items with 3 subscales: clinical situations, internal constraints, and external constraints. *Types of stressors measured*: Workload/time pressure, Lack of participation in workplace and service-level policies, Lack of decision authority/autonomy (timing, method, etc.), Lack of assertiveness when confronted with ethical concerns, Observing deviations from safety standards, Unsuitability of care (Futility or over/under aggressiveness of therapeutics), Working with incompetent/inexperienced /negligent staff members, Ignoring patients' preferences and conditions, Unsafe orders/policies from hierarchies or persons in charge
Nursing Stress Scale (NSS)	8 of which 2 used with another scale	(17, 25, 26, 53, 54, 61, 65, 75)	Type 1	Healthcare settings	Nurses only	34 items with 7 subscales: death and suffering of patients, conflict with doctors, lack of appropriate training, lack of social support, conflict with other nurses, excessive workload, and uncertainty about the treatment carried out. *Types of stressors measured*: Lack of positive/supportive relationship with colleagues, Conflict with colleagues, Death and suffering and emotion regulation, Communicating with and fulfilling the emotional needs of users or relatives, Conflict with supervisor, Workload/time pressure, Lack of task-related skills or preparation (incl. training, knowledge update), Lack of a predictable, stable, and recovery-propitious schedule, Lack of staffing, Lack of information (e.g., to ask patients' questions), Lack of/or low-quality or low accessibility to material resources, Lack of predictable and stable work relationships and places, Risk of making severe errors
Nursing Work Index (NWI) and variants (PES-NWI/NWI EO/NWI-R)	6 of which 2 used with another scale and 1 not used entirely	(7, 12, 48, 56, 67, 77)	Type 1 (5 articles) Type 2 (1 article)	Healthcare settings	Nurses only	31 to 57 items with 5 subscales: nurse participation in hospital affairs, nursing foundations for quality of care, nurse manager, ability, leadership and support of nurses, staffing and resource adequacy, collegial nurse physician relations. *Types of stressors measured*: Lack of positive/supportive relationship with colleagues, Lack of instrumental support from colleagues, Lack of positive/supportive relationship with supervisor, Lack of team cohesion, Lack of instrumental support from supervisor, Lack of or inappropriate inter-services/administrative collaboration, Lack of value-based team harmony/tolerance, Interruption/interference/distraction/unanticipated changes and resequencing, Lack of participation in workplace and service-level policies, Lack of decision authority/autonomy (timing, method, etc.), Lack of growth opportunities, Lack of a preceptor program for newly hired personnel, Lack of a predictable, stable, and recovery-propitious schedule, Lack of staffing, Lack of information (e.g., unable to question patients?), Lack of predictable and stable work relationships and place, Working with incompetent/inexperienced/negligent staff members
Nurse Stress Index (NSI)	4 of which 1 used with another scale	(9, 47, 52, 70)	Type 1	Healthcare settings	Nurses only	30 items with 6 subscales: Workload (time management), Workload (managerial demands), lack of organizational support, Work–Home conflict, Confidence and Competence, Dealing with difficult patients and relatives. *Types of stressors measured*: Lack of instrumental support from colleagues, Lack of instrumental support from supervisor, Death and suffering and emotion regulation, Communicating with and fulfilling the emotional needs of users or relatives, Conflict with inappropriate expectations or behaviors from users (customer, client, patient, etc.), Conflict with inappropriate expectations or behaviors from users' relatives, Workload/time pressure, Lack of task-related skills or preparation (incl. training, knowledge update), Work–Home conflict (e.g., because of night shifts, on site call)
Hospital Survey Of Patient Safety Culture (HSOPSC)	2	(96, 97)	Type 1	Healthcare settings	Healthcare professionals	42 items with 12 subscales: supervisor expectations/actions promoting patient safety, organizational learning continuous improvement, teamwork, communication openness, feedback and error reporting, nonpunitive response to error, staffing, hospital management toward patient safety, teamwork across the hospital, handoffs and transitions, general perception of patient safety, frequency of errors reporting. *Types of stressors measured*: Lack of positive/supportive relationship with colleagues, Lack of instrumental support from colleagues, Lack of team cohesion, Lack of or inappropriate inter-services/administrative collaboration, Workload/time pressure, Lack of participation in workplace and service-level policies, Lack of staffing, Lack of information (e.g., unable to question patients), Observing deviations from safety standards, Unsafe orders/policies from hierarchies or person in charge
Tummers (2002, 2005) Organizational and work characteristics questionnaire	2	(88, 89)	Type 4 (A)	Healthcare settings	Healthcare professionals	Number of items unavailable. *Types of stressors measured*: Lack of positive/supportive relationship with colleagues, Lack of instrumental support from colleagues, Lack of positive/supportive relationship with supervisor, Lack of instrumental support from supervisor, Conflict with colleagues, Conflict with supervisor, Workload/time pressure, Interruption/interference/distraction/unanticipated changes and resequencing, Task complexity/High level of attention/performance, Lack of decision authority/autonomy (timing, method, etc.), Constant alert and sudden emergencies due to the patient's condition
Sagie and Krausz (2003) scheduling control scale	1 used with another scale	(78)	Type 3 (A)	Healthcare settings	Healthcare professionals	Number of items unavailable. Nurses' perceptions of having choice and influence on the timing and scheduling of their work. *Types of stressors measured*: Lack of participation in workplace and service-level policies, Lack of decision authority/autonomy (timing, method, etc.)
Job Stress Scale (JSS)	1	(8)	Type 1	Healthcare settings	Healthcare professionals	22 items with 4 subscales: competence, physical work environment, staffing, team respect. *Types of stressors measured*: Lack of positive/supportive relationship with colleagues, Taxing work environment (noisy, hectic, crowded, heated, etc.), Lack of task-related skills or preparation (incl. training, knowledge update), Lack of staffing
Work-Related Strain Inventory (WRSI)	1	(4)	Type 1	Healthcare settings	Healthcare professionals	18 items with no identified subscales. *Types of stressors measured*: Lack of positive/supportive relationship with colleagues, Lack of positive/supportive relationship with supervisor, Conflict with colleagues, Conflict with supervisor, Workload/time pressure
Nursing Job Stressor Scale (NJSS)	1	(45)	Type 1	Healthcare settings	Nurses only	33 items with 7 subscales: Conflict with other nursing staffs, nursing role conflict, conflict with physicians/autonomy, dealing with death and dying, qualitative work load, quantitative work load and conflict with patients. *Types of stressors measured*: Lack of positive/supportive relationship with colleagues, Lack of instrumental support from supervisor, Conflict with colleagues, Death and suffering and emotion regulation, Communicating with and fulfilling the emotional needs of users or relatives, Conflict with inappropriate expectations or behaviors from users (customer, client, patient, etc.), Workload/time pressure, Task complexity/High level of attention/performance, Lack of task-related skills or preparation (incl. training, knowledge update), Lack of staffing, Lack of information (e.g., to ask patients' questions), Risk of making severe errors
Modified version of Cooper's Job Stress Questionnaire (CJSQ)	1	(66)	Type1	Healthcare settings	Healthcare professionals	16 items with no identified subscales. *Types of stressors measured*: Death and suffering and emotion regulation, Communicating with and fulfilling the emotional needs of users or relatives, Assault/aggression from patients or relatives, Lack of patients' recognition, Workload/time pressure, Interruption/interference/distraction/unanticipated changes and resequencing, Taxing work environment (noisy, hectic, crowded, heated, etc.), Risk of making severe errors, Constant alert and sudden emergencies due to the patient's condition, Work–Home conflict (e.g., because of night shifts, on site call)
Nine Equivalent of nursing Manpower use (NEMS)	1 used with another scale	(6)	Type 1	Healthcare settings	Nurses only	9 items with no subscales. *Types of stressors measured*: Workload/time pressure, Task complexity/High level of attention/performance
Hospital Ethical Climate Scale (HECS-S)	1 used with another scale	(98)	Type 1	Healthcare settings	Healthcare professionals	26 items with 5 subscales: relation between colleagues, patients, managers, hospital, and physicians. *Types of stressors measured*: Lack of positive/supportive relationship with colleagues, Lack of instrumental support from colleagues, Lack of positive/supportive relationship with supervisor, Lack of team cohesion, Lack of instrumental support from supervisor, Lack of or inappropriate inter-services/administrative collaboration, Lack of participation in workplace and service-level policies, Lack of information (e.g., unable to question patients), Observing deviations from safety standards, Working with incompetent/inexperienced/negligent staff members, Ignoring patients' preferences and conditions
Environmental Complexity Scale	1 used with another scale	(67)	Type 1	Healthcare settings	Healthcare professionals	33 items with 3 subscales: Change of acuity, Resequencing, Team. *Type of stressors measured*: Interruption/interference/distraction/unanticipated changes and resequencing
Varjus et al. (2003) Nurses' Autonomy Scale	1 used with another scale	(68)	Type 1	Healthcare settings	Nurses only	18 items with 3 subscales: knowledge, action, value bases of autonomy. *Types of stressors measured*: Lack of participation in workplace and service-level policies, Lack of decision authority/autonomy (timing, method, etc.)
Verhaeghe et al. (2008) negative appraisal of recurrent changes in the work environment	1 used with another scale	(93)	Type 4 (A)	Healthcare settings	Healthcare professionals	6 items with no identified subscales. *Types of stressors measured*: Lack of a predictable, stable, and recovery-propitious schedule, Lack of predictable and stable work relationships and places
Vessey et al. (2009) Nurses bullying questionnaire	1	(94)	Type 4 (A)	Healthcare settings	Nurses only	30 items with no identified subscales. *Type of stressors measured*: Injustice, discrimination, harassment, bullying
Casado et al. (2008) Occupational factors questionnaire	1	(10)	Type 4 (A)	Healthcare settings	Healthcare professionals	Number of items unavailable. *Types of stressors measured*: Lack of positive/supportive relationship with colleagues, Lack of positive/supportive relationship with supervisor, Workload/time pressure, Lack of a predictable, stable, and recovery-propitious schedule
Amin et al. (2009) potential stressors questionnaire	1	(2)	Type 5	Healthcare settings	Healthcare professionals	6 items with no identified subscales. *Types of stressors measured*: Lack of positive/supportive relationship with colleagues, Lack of instrumental support from colleagues, Lack of instrumental support from supervisor, Workload/time pressure, Lack of a predictable, stable, and recovery-propitious schedule, Lack of job security, Insufficient remuneration
Fujita et al. (2011) workplace violence questionnaire	1	(23)	Type 5	Healthcare settings	Healthcare professionals	3 questions about workplace violence, the work environment, and other topics. *Type of stressors measured*: Assault/aggression from patients or relatives
Safety Attitudes Questionnaire–ICU (SAQ-ICU)	6	(32, 37, 50, 72, 74, 102)	Type 1	ICU settings	Healthcare professionals	32 items with 6 subscales: teamwork climate, safety climate, job satisfaction, stress recognition, perception of management, working conditions. *Types of stressors measured*: Lack of positive/supportive relationship with colleagues, Lack of instrumental support from colleagues, Lack of positive/supportive relationship with supervisor, Lack of team cohesion, Lack of instrumental support from supervisor, Lack of or inappropriate inter-services/administrative collaboration, Lack of participation in workplace and service-level policies, Lack of staffing, Lack of information (e.g., to ask patients' questions), Observing deviations from safety standards, Working with incompetent/inexperienced/negligent staff members, Unsafe orders/policies from hierarchies or person in charge
Embriaco et al. (2007a) Working conditions questionnaire	4 of which 1 used with another scale	(21, 22, 59, 84)	Type 4 (A)	ICU settings	Healthcare professionals	Number of items unavailable. *Types of stressors measured*: Conflict with colleagues, Death and suffering and emotion regulation, Conflict with supervisor, Conflict with inappropriate expectations or behaviors from users (customer, client, patient, etc.), Conflict with inappropriate expectations or behaviors from users' relatives, Workload/time pressure, Lack of a predictable, stable, and recovery-propitious schedule, Decisional dilemmas/uncertainty regarding patients' survival and end-of-life care, Risk of making severe errors
Teixeira et al. (2014) Ethical decision experiences of IC professionals	2 of which 1 used with another scale	(83, 84)	Type 4 (A)	ICU settings	Healthcare professionals	Number of items unavailable. *Type of stressors measured*: Decisional dilemmas/uncertainty regarding patients' survival and end-of-life care
Coomber et al. (2002) ICU-related stressors questionnaire	2	(14, 91)	Type 4	ICU settings	Healthcare professionals	30 items with no identified subscales. *Types of stressors measured*: Lack of positive/supportive relationship with colleagues, Lack of positive/supportive relationship with supervisor, Lack of team cohesion, Communicating with and fulfilling the emotional needs of users or relatives, Conflict with supervisor, Lack of or inappropriate inter-services/administrative collaboration, Workload/time pressure, High managerial/decisional responsibilities, Lack of a predictable, stable, and recovery-propitious schedule, Lack of or low-quality or low accessibility to material resources, Decisional dilemmas/uncertainty regarding patients' survival and end-of-life care, Observing deviations from safety standards, Unsuitability of care (Futility or over/under aggressiveness of therapeutics, Risky situations for oneself, Work–Home conflict (e.g., because of night shifts, on site call)
ICU nursing stress audit	1	(27)	Type 1	ICU settings	Nurses only	47 items with 9 subscales: management, interpersonal, relationships, patient care, knowledge, skill, work environment, self-perception, and administrative uncertainties. *Types of stressors measured*: Lack of positive/supportive relationship with colleagues, Lack of instrumental support from colleagues, Lack of positive/supportive relationship with supervisor, Lack of team cohesion, Conflict with colleagues, Death and suffering and emotion regulation, Communicating with and fulfilling the emotional needs of users or relatives, Lack of or inappropriate inter-services/administrative collaboration, Workload/time pressure, Interruption/interference/distraction/unanticipated changes and resequencing, Taxing work environment (noisy, hectic, crowded, heated, etc.), High managerial/decisional responsibilities, Lack of growth opportunities, Lack of task-related skills or preparation (incl. training, knowledge update), Lack of a predictable, stable, and recovery-propitious schedule, Lack of staffing, Lack of adequate rules and procedures, Lack of task meaning/utility, Unsuitability of care (Futility or over/under aggressiveness of therapeutics), Working with incompetent/inexperienced/negligent staff members, Risk of making severe errors, Constant alert and sudden emergencies due to the patient's condition, Lack of job security, Insufficient remuneration
Piers et al. (2011a) Perceived ethical environment questionnaire	1 used with another scale	(71)	Type 3 (A)	ICU settings	Healthcare professionals	7 items with no identified subscales. *Types of stressors measured*: Lack of positive/supportive relationship with colleagues, Lack of team cohesion, Lack of value-based team concordance /tolerance, Lack of participation in workplace and service-level policies
Hays et al. (2006) list of 13 stressors in ICU	1	(35)	Type 3 (A, B)	ICU settings	Healthcare professionals	13 items with no identified subscales. *Types of stressors measured*: Death and suffering and emotion regulation, Lack of or inappropriate inter-services/administrative collaboration, Conflict with inappropriate expectations or behaviors from users' relatives, Taxing work environment (noisy, hectic, crowded, heated, etc.), Lack of staffing, Unsuitability of care (Futility or over/under aggressiveness of therapeutics), Working with incompetent/inexperienced/negligent staff members, Risk of making severe errors, Constant alert and sudden emergencies due to the patient's condition
Comprehensive Nursing Intervention Score (CNIS)	1	(101)	Type 3 (A)	ICU settings	Nurses only	73 items with 8 subscales: Monitoring, Transfusion of blood/fluids, Injections, Respiratory management, Assisted circulation, Drainage tube management, Special therapy, Basic nursing care. *Types of stressors measured*: Communicating with and fulfilling the emotional needs of users or relatives, Workload/time pressure, Task complexity/High level of attention/performance, Physical efforts during tasks performance
Azoulay et al. (2009) Nurse-physician conflicts questionnaire	1 used with another scale	(5)	Type 3 (B)	ICU settings	Healthcare professionals	Number of items unavailable, 3 subscales: parties involved in the conflict, source of the conflict, and clinical impact and severity of the conflict. *Types of stressors measured*: Lack of positive/supportive relationship with colleagues, Lack of instrumental support from colleagues, Lack of positive/supportive relationship with supervisor, Lack of team cohesion, Lack of instrumental support from supervisor, Conflict with colleagues, Conflict with supervisor, Lack of or inappropriate inter-services/administrative collaboration, Role conflicts/contradictory demands, Lack of participation in workplace and service-level policies, Lack of staffing, Observing deviations from safety standards, Unsuitability of care (Futility or over/under aggressiveness of therapeutics), Working with incompetent/inexperienced/negligent staff members, Ignoring patients' preferences and conditions
Well-Being of Intensive Care nurses (WEBIC)-questionnaire	1	(49)	Type 3 (A)	ICU settings	Nurses only	Number of items unavailable. *Types of stressors measured*: Workload/time pressure, Task complexity/High level of attention/performance
Van dam et al. (2012) Scale	1	(90)	Type 4 (A)	ICU settings	Healthcare professionals	38 items with 10 subscales. Dealing with night shifts, Technical orientation, Emotional demands, Physical demands, Threats from relatives, Social support, Autonomy, Development opportunities, Work pressure and Turnover intention. *Types of stressors measured*: Conflict with inappropriate expectations or behaviors from users' relatives, Lack of task diversity/interest, Lack of a predictable, stable, and recovery-propitious schedule
Hansen et al. (2009) Perceptions of end-of-life care factors	1	(34)	Type 4 (A)	ICU settings	Healthcare professionals	30 items with 5 subscales: knowledge and ability, work environment, support for staff, support for patients and patients' families, and work stress related to specific end-of-life situations. *Types of stressors measured*: Lack of positive/supportive relationship with colleagues, Lack of instrumental support from colleagues, Lack of positive/supportive relationship with supervisor, Lack of team cohesion, Lack of instrumental support from supervisor, Communicating with and fulfilling the emotional needs of users or relatives, Lack of task-related skills or preparation (incl. training, knowledge update), Lack of information (e.g., to ask patients' questions), Lack of or low-quality or low accessibility to material resources, Decisional dilemmas/uncertainty regarding patients' survival and end-of-life care, Unsuitability of care (Futility or over/under aggressiveness of therapeutics), Ignoring patients' preferences and conditions, Lack of participation in end-of-life decisions or strong disagreement
End-of-lIfe DECision-making and staff Stress (EIDECS) questionnaire	1	(80)	Type 4	ICU settings	Healthcare professionals	37 items with no identified subscales. *Types of stressors measured*: Lack of positive/supportive relationship with colleagues, Lack of positive/supportive relationship with supervisor, Lack of team cohesion, Death and suffering and emotion regulation, Lack of or inappropriate inter-services/administrative collaboration, Workload/time pressure, Interruption/interference/distraction/unanticipated changes and resequencing, Lack of participation in workplace and service-level policies, Lack of information (e.g., to ask patients' questions), lack of task–role clarity
Questionnaire of Performance Obstacles of Intensive Care Nurses (QPO-ICN)	1	(63)	Type 4	ICU settings	Nurses only	53 items with no identified subscales. *Type of stressors measured*: Lack of instrumental support from colleagues
Piers et al. (2011) (b) Perceived Inappropriateness of Care Questionnaire	1 used with another scale	(71)	Type 4	ICU settings	Healthcare professionals	7 items with no subscales. *Types of stressors measured*: Observing deviations from safety standards, Unsuitability of care (Futility or over/under aggressiveness of therapeutics), Ignoring patients' preferences and conditions, Lack of participation in end-of-life decisions or strong disagreement, Inaccurately informed patients and families
Hamric and Blackhall (2007)	1 used with another scale	(33)	Type 4 (A)	ICU settings	Healthcare professionals	Number of items unavailable, 4 subscales: ethical environment, end-of-life communication, satisfaction with quality of care, collaboration. *Types of stressors measured*: Lack of instrumental support from colleagues, Lack of team cohesion, Decisional dilemmas/uncertainty regarding patients' survival and end-of-life care, Observing deviations from safety standards
Malaquin et al. (2016) well-being at work	1	(58)	Type 5	ICU settings	Healthcare professionals	Number of items unavailable. *Types of stressors measured*: Lack of positive/supportive relationship with colleagues, Lack of instrumental support from colleagues, Lack of positive/supportive relationship with supervisors, Lack of instrumental support from supervisors, Conflict with colleagues, Conflict with supervisors, Workload/time pressure, Lack of a predictable, stable, and recovery-propitious schedule, Finding time for research
Kincey et al. (2010) Potential sources of pressure in ICM and healthcare settings	1	(44)	Type 5	ICU settings	Healthcare professionals	40 items with no identified subscales. *Types of stressors measured*: Lack of team cohesion, Workload/time pressure, Lack of a predictable, stable, and recovery-propitious schedule, Lack of staffing, Lack of or low-quality or low accessibility to material resources, Lack of predictable and stable work relationships and places, Decisional dilemmas/uncertainty regarding patients' survival and end-of-life care, Observing deviations from safety standards, Risk of making severe errors, Constant alert and sudden emergencies due to patients' conditions, Work–Home conflict (e.g., because of night shifts, on site call)
Grzeskowiak et al. (2012) 10 most common real situations in the PICU	1	(31)	Type 5	ICU settings	Healthcare professionals	10 items with no identified subscales. *Types of stressors measured*: Death and suffering and emotion regulation, Communicating with and fulfilling the emotional needs of users or relatives, Supervisor's evaluation, Decisional dilemmas/uncertainty regarding patients' survival and end-of-life care, Risk of making severe errors
Poncet et al. (2007) work-related factors questionnaire	1	(73)	Type 5	ICU settings	Healthcare professionals	23 items with no identified subscales. *Types of stressors measured*: Conflict with colleagues, Death and suffering and emotion regulation, Conflict with supervisor, Conflict with inappropriate expectations or behaviors from users (customer, client, patient, etc.), Conflict with inappropriate expectations or behaviors from users' relatives, Lack of participation in workplace and service-level policies, Lack of a predictable, stable, and recovery-propitious schedule, Decisional dilemmas/uncertainty regarding patients' survival and end-of-life care
Shehabi et al. (2009) Self-reported 12-weekly averaged workload pattern	1	(81)	Type 5	ICU settings	Healthcare professionals	Number of items unavailable. *Type of stressors measured*: Workload/time pressure
Janda et Jandovà (2015)	1	(38)	Type 5	ICU settings	Healthcare professionals	15 items no subscales identified. *Types of stressors measured*: Conflict with colleagues, Death and suffering and emotion regulation, Communicating with and fulfilling the emotional needs of users or relatives, Conflict with inappropriate expectations or behaviors from users' relatives, Lack of staffing, Decisional dilemmas/uncertainty regarding patients' survival and end-of-life care, Unsuitability of care (Futility or over/under aggressiveness of therapeutics), Constant alert and sudden emergencies due to the patient's condition, Risky situations for oneself

*Type 1: Ante hoc validated scales*.

*Type 2: Ante hoc validated scales with ad hoc modifications*.

*Type 3: Ad hoc scales with validity-related information on item origin and basic statistics*.

*Type 4: Ad hoc scales with validity-related information on item origin or basic statistics*.

*Type 5: Ad hoc scales without any validity-related information*.

*(A): Ad hoc items derived from literature and/or another scale*.

*(B): Ad hoc items derived from interviews or conference consensus with healthcare professionals*.

Overall, among the 102 studies seeking to identify stressors in the ICU, 36 used a more or less problematic scale in terms of validity (Types 2–5).

### Types of Stressors Assessed in Intensive Care Unit Studies

Eight major types of stressors, grouping 58 subtypes, have been identified (see Table 2; for the coding of each scale, see Table 1): (1) *High job demands* (40 scales); (2) *Problematic relationships with other professionals* (39 scales); (3) *Lack of control over work situations and career* (31 scales); (4) *Lack of organizational resources* (29 scales); (5) *Problematic situations with users and relatives* (20 scales); (6) *Dealing with ethical and moral-related situations* (19 scales); (7) *Risk management issues* (14 scales); (8) *Disadvantages in comparison to other occupational situations* (11 scales). Two types of stressors do not fall within these eight main categories and have been classified as “other” (measured by three different scales).

**Table 2:** Broad types and subtypes of stressors measured by each type of scales used in ICU studies.

**Broad types of stressors**	**Subtypes of stressors**	**All scales (*****N*** **=** **59)**		**All settings scales (*****n*** **=** **17)**		**Healthcare settings scales** **(*****n*** **=** **20)**		**ICU settings scales (*****n*** **=** **22)**	
		***N***	**%**	***N***	**Corrected %^**a**^**	***N***	**Corrected %^**a**^**	***N***	**Corrected %^**a**^**
*High job demands* 40 scales with at least one subtype	1. Workload /time pressure	31	53	11	40	11	34	9	25
	2. Interruption/interference/distraction/unanticipated changes and resequencing	12	20	4	38	5	40	3	22
	3. Task complexity/High level of attention/performance	11	19	6	59	3	25	2	15
	4. Taxing work environment (noisy, hectic, crowded, heated, etc.)	9	15	3	38	3	32	3	29
	5. Role conflicts/contradictory demands	8	14	7	90	0	0	1	10
	6. High managerial/decisional responsibilities	5	8	3	66	0	0	2	34
	7. Physical efforts during task performance	4	7	3	80	0	0	1	20
	8. Underload	1	2	1	100	0	0	0	0
	TOTAL	81	18	38	52	22	26	21	22
*Problematic relationships with other professionals* 39 scales with at least one subtype	1. Lack of positive/supportive relationship with colleagues	25	42	7	32	10	39	8	29
	2. Lack of instrumental support from colleagues	19	32	6	36	6	31	7	33
	3. Lack of positive/supportive relationship with supervisor	17	29	4	28	6	35	7	37
	4. Lack of team cohesion	15	25	3	24	3	20	9	56
	5. Lack of instrumental support from supervisor	14	24	4	33	6	42	4	25
	6. Conflict with colleagues	14	24	4	33	4	28	6	39
	7. Conflict with supervisor	12	20	4	38	3	24	5	37
	8. Lack of/or inappropriate inter-services/administrative collaboration	11	19	1	11	3	28	7	60
	9. Injustice, discrimination, harassment, bullying	4	7	3	78	1	22	0	0
	10. Lack of value-based team concordance /tolerance	2	3	0	0	1	52	1	48
	11. Supervisor's evaluation	1	2	0	0	0	0	1	100
	TOTAL	134	33	36	31	43	32	55	37
*Lack of control over work situations and career* 31 scales with at least one subtype	1. Lack of participation to workplace and service-level policies	14	24	3	25	6	43	5	32
	2. Lack of decision authority/autonomy (timing, method, etc.)	12	20	7	62	5	38	0	0
	3. Lack of growth opportunities	7	12	5	75	1	13	1	12
	4. Lack of tasks-related skills or preparation (incl. training, knowledge update)	7	12	1	17	4	57	2	26
	5. Skill underutilization	6	10	6	100	0	0	0	0
	6. Lack of task diversity/interest	4	7	3	80	0	0	1	20
	7. Lack of a preceptor program for newly hired personnel	1	2	0	0	1	100	0	0
	8. Lack of assertiveness in front of ethical concerns	1	2	0	0	1	100	0	0
	TOTAL	52	12	25	53	18	32	9	15
*Lack of organizational resources* 29 scales with at least one subtype	1. Lack of a predictable, stable, and recovery-propitious schedule	14	24	2	17	5	36	7	46
	2. Lack of staffing	12	20	1	10	5	43	6	47
	3. Lack of information (e.g., to ask patients' questions)	11	19	2	21	5	45	4	33
	4. Lack of/or low-quality or low accessibility to material resources	7	12	2	34	1	14	4	52
	5. Lack of predictable and stable work relationships and place	5	8	1	23	3	59	1	18
	6. lack of task–role clarity	5	8	4	84	0	0	1	16
	7. Lack of adequate rules and procedures	3	5	1	39	0	0	2	61
	8. Lack of task meaning/utility	3	5	2	72	0	0	1	28
	9. Lack of hierarchical role clarity	2	3	2	100	0	0	0	0
	TOTAL	62	14	17	32	19	30	26	38
*Problematic situations with service users and relatives* 20 scales with at least one subtype	1. Death and suffering and emotion regulation	13	22	2	19	4	31	7	50
	2. Communicating with and fulfilling emotional needs of users or relatives	12	20	2	20	4	34	6	46
	3. Conflict with inapropriate expectations or behaviors from users (customer, client, patient, etc.)	7	12	2	33	3	42	2	25
	4. Conflict with inapropriate expectations or behaviors from users' relatives	6	10	0	0	1	18	5	82
	5. Assault/aggression from users or relatives	2	3	0	0	2	100	0	0
	6. Lack of users' recognition	1	2	0	0	1	100	0	0
	TOTAL	41	10	6	18	15	37	20	45
*Dealing with ethical and moral-related situations* 19 scales with at least one subtype	1. Decisional dilemmas/uncertainty regarding patients' survival and end-of-life care	10	17	0	0	0	0	10	100
	2. Observing deviations from safety standards	10	17	1	12	3	31	6	57
	3. Unsuitability of care (Futility or over/under aggressiveness of therapeutics)	8	14	0	0	1	14	7	86
	4. Working with incompetent/unexperienced/negligent staff members	7	12	0	0	3	45	4	55
	5. Ignoring patient's preferences and conditions	5	8	0	0	2	42	3	58
	6. Unsafe orders/policies from hierarchies or person in charge	3	5	0	0	2	69	1	31
	7. Lack of participation in end-of-life decisions/or strong disagreement	2	3	0	0	0	0	2	100
	8. Inaccurately informed patients and families	1	2	0	0	0	0	1	100
	TOTAL	46	10	1	3	11	26	34	72
*Risk management issues* 14 scales with at least one subtype	1. Risk of making severe errors	9	15	1	13	3	34	5	52
	2. Constant alert and sudden emergencies due to the patient's condition	6	10	0	0	2	35	4	65
	3. Risky situations for oneself	5	8	3	66	0	0	2	34
	TOTAL	20	4	4	24	5	25	11	51
*Job disadvantages in comparison to other occupational situations* 11 scales with at least one subtype	1. Lack of job security	7	12	5	75	1	13	1	12
	2. Work–Home conflict (e.g., because of night shifts, on site call)	6	10	2	38	2	32	2	29
	3. Insufficient remuneration	3	5	1	38	1	32	1	29
	TOTAL	16	4	8	55	4	23	4	21
*Other*	1. Making time for research	2	3	0	0	0	0	2	100
3 scales with at least one subtype	2. Lack of pride/self-respect	1	2	1	100	0	0	0	0

a *The correction accounts for the unequal numbers of scales of each type (17, 20, and 22) to allow a valid comparison of row percentages. The formula was (N scales of the target type that measured the stressor/Overall N scales that measured the stressor)/((N all settings scales that measured the stressor/17) + (N healthcare settings scales that measured the stressor/20) + (N ICU settings scales that measured the stressor/22))*.

The *High job demands* category refers to taxing task-, workflow-, and role-related situations and distinguishes between eight subtypes. The *problematic relationships with other professionals* category includes 11 subtypes of stressors related to the lack of support, communication/collaboration problems, or conflicts with colleagues, superiors, or other departments. The *lack of control over work situations and career* category includes the different control and skill-related factors which may prevent professionals from being responsive, efficient, and committed. This category of stressors includes eight subtypes. The *lack of organizational resources* category includes nine subtypes of resources the healthcare organization might fail to sufficiently provide to promote the quality of life and the quality of care of ICU professionals. The *problematic situations with service users and relatives* category includes six subtypes of stressors which all involve one or more people whose behavior or health condition is likely to exceed the resources of healthcare professionals. The *dealing with ethical- and moral-related situations* category refers to eight subtypes of situations where patients' well-being and safety are likely to be harmed by unsuitable decisions/actions of the ICU team. The *risk management issues* category refers to risky situations for both patients and professionals with a substantial probability of making critical errors (three subtypes included). Lastly, the *disadvantages in comparison to other occupational situations* category of stressors concerns the extent to which the professional considers his/her current occupational situation is disadvantaged in comparison to other career choices (e.g., other jobs, other employers, other professions) (three measured subtypes).

### Ability of Scales to Take Into Account the Specificity of Intensive Care Unit Stressors

There are important differences concerning the extent to which the three types of scales (i.e., all settings, healthcare settings, and ICU settings scales) address the broad range of the types and subtypes of stressors mentioned above. To highlight these differences, we compared the proportion of each type of scale that measured a given stressor relative to the overall number of scales that measured it. However, given that there were more ICU settings scales (*N* = 22) than healthcare settings scales (*N* = 20) and all settings scales (*N* = 17), we applied a correction to make the comparisons valid^1^.

**Figure 2:**
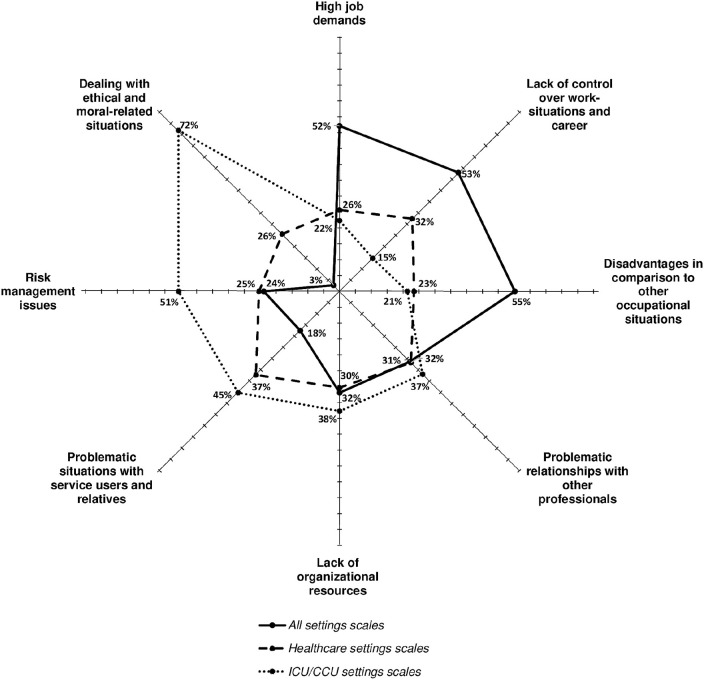
Comparison of the three types of scales regarding their propensity to measure the eight broad types of stressors (% is the corrected proportion of scales of a type covering at least one stressor in the target category).

As illustrated by Figure 2, *high job demands* (52%), *disadvantages in comparison to other occupational situations* (55%), and *lack of control over work situations and career* (53%) were more frequently measured by the all settings scales than by the other two scales (ranging from 15 to 32%). However, all settings scales were rarely used to measure *risk management issues* (24%) and *problematic situations with service users and relatives* (18%) and were hardly used to measure *dealing with ethical- and moral-related situations* (3%); conversely, ICU settings scales were used more frequently to measure these broad types of stressors (51, 45, and 72%, respectively). Healthcare settings scales were used to measure *problematic situations with service users and relatives* (37%) almost as often as ICU settings scales, but less frequently to measure *risk management issues* (25%) and *dealing with ethical- and moral-related situations* (26%). The three types of scales measured *lack of organizational resources* (30–38%) and *problematic relationships with other professionals* (31–37%) to the same extent.

Beyond these eight broad types of stressors, there were a number of important differences concerning their subtypes. For instance, some stressors were more frequently measured by ICU scales than by the other two scales: decisional dilemmas/uncertainty regarding patients' survival and end-of-life care (100%), unsuitability of care (futility or over/under aggressiveness of therapeutics) (86%), conflict and inappropriate expectations or behavior from relatives (82%), or constant alert and sudden emergencies due to the patient's condition (65%).

In addition, in terms of item content, ICU settings scales and, to a lesser extent, healthcare settings scales generally refer more to precise and tailored features of the working environment of ICU professionals than all settings scales.

### Scales Most Frequently Used in Intensive Care Unit Studies and Comparison of Their Metrological Properties and Specificities

Approximately two thirds of the retrieved studies used at least one of the eight following scales (out of the 59 scales identified) (Table 1): 17 studies used the Job Content Questionnaire (JCQ) or one of its variants (Karasek et al., 1998; Piers et al., 2011); 15 studies used the Moral Distress Scale (MDS) or one of its variants (Corley et al., 2001; Hamric et al., 2012); eight studies used the Nursing Stress Scale (NSS) (Gray-Toft and Anderson, 1981); six studies used the Safety Attitudes Questionnaire–ICU version (SAQ-ICU) (Sexton et al., 2006); six studies used the Nursing Work Index (NWI) or one of its variants (Kramer and Hafner, 1989; Aiken and Patrician, 2000; Lake, 2002; Bonneterre et al., 2011); four studies used the Nurses Stress Index (NSI) (Harris, 1989); four studies used the scale of Embriaco et al. (2007a); and three studies used the Effort–Reward Imbalance (ERI) questionnaire (Siegrist et al., 2004). Other scales such as the Daily Hassles Questionnaire (DHQ) (Kanner et al., 1981), Workplace Stress Scale (WSS) (The Marlin Company and the American Institute of Stress, 2011), Hospital Survey Of Patient Safety Culture (HSOPSC) (Sorra and Dyer, 2010), Copenhagen Psychosocial Questionnaire V2 (COPSOQ2) (Pejtersen et al., 2010), ICU-related stressors questionnaire (Coomber et al., 2002), and the scales of Tummers et al. (2002) or Teixeira et al. (2013) were used in two studies each. Finally, the 44 other scales were used in one article each, including 10 all settings scales (four Type 1 and four Type 2 scales, Rizzo et al., 1970; Kanner et al., 1978; Cooper and Williams, 1991; Dhondt and Houtman, 1992; Jackson et al., 1993; Veldhoven and Meijman, 1994; Semmer et al., 1995; Shimomitsu et al., 2000; Stinglhamber and Vandenberghe, 2003; Hart, 2006), 15 healthcare settings scales (10 Type 1 scales, Hinshaw and Atwood, 1985; Revicki et al., 1991; Miranda et al., 1997; O'Brien-Pallas et al., 1997; Tyssen et al., 2001; Kitaoka-Higashiguchi and Nakagawa, 2003; Varjus et al., 2003; Olson, 2007; Einarsen et al., 2009; one Type 3, three Type 4, and two Type 5 scales), and 19 ICU settings scales (one Type 1, Bailey et al., 1980; five Type 3, six Type 4, and six Type 5 scales).

An analysis of the 59 scales reveals that no scale covered all the main stressors in ICUs (Table 2), not even those with satisfactory metrological properties. For instance, the JCQ, the most used across the world, is a Type 1 scale (*ante hoc* with satisfying validity-related statistics) which has primarily been used to measure the following types of stressors: *lack of control over work situations and career, high job demands*, and *problematic relationships with other professionals*. However, this scale does not cover stressors such as *dealing with ethical- and moral-related situations, problematic situations with service users and relatives*, and *risk management issues* and measures only one stressor in the broad category of the *lack of organizational resources* (note: the full 49-item JCQ also measures Lack of job security, a stressor associated with the *disadvantages in comparison with other occupational situations* category, but this version was not used in the retrieved ICU studies). Furthermore, four of the 17 studies retrieved combined it with a healthcare settings scale [NSS, NWI, Nine Equivalent of nursing Manpower use (NEMS)] or an ICU settings scale such as the “Perceived ethical environment questionnaire” and the “Perceived inappropriateness of care questionnaire” developed by Piers et al. (2011).

The second most frequently used scale in ICU studies—the MDS—also presents certain limitations regarding its coverage of the different types of stressors. It is a Type 1 scale, but it is tailored to measure stressors relevant in many healthcare settings which focus primary on stressors related to *dealing with ethical- and moral-related situations*. It also measures stressors relating to the *lack of control over work situations and career*, as well as one stressor relating to *high job demands*. However, this scale does not measure stressors relating to the following categories: *problematic relationships with other professionals, problematic situations with service users and relatives, lack of organizational resources, risk management issues*, and *disadvantages in comparison to other occupational situations*. This led authors of two out of the 16 studies which used the MDS to combine it with either a healthcare settings scale (Varjus et al., 2003) or an *ad hoc* ICU-specific questionnaire (Hamric and Blackhall, 2007) to measure additional stressors.

Similar observations can be made with regard to the third most frequently used scale, i.e., the NSS. This Type 1 scale specifically targets nurses (i.e., it is unsuitable for other healthcare professions). It primarily covers stressors from four broad types: *high job demands, lack of organizational resources, problematic relationships with other professionals*, and *problematic situations with service users and relatives*. However, it measures one stressor from the *lack of control over work situations and career* category to a limited extent and none from the categories *dealing with ethical- and moral-related situations, risk management issues*, and *disadvantages in comparison with other occupational situations*.

The same comments were made for all other scales. No scale covered the eight broad types of stressors in a comprehensive manner, and many of them were problematic in terms of validity/reliability. It is worth mentioning that 14 articles analyzed combined two or three scales to increase stressor coverage.

## Discussion

From the 102 studies analyzed, we identified 59 different scales. Only 28 out of the 59 scales were validated (Type 1), and two ICU settings scales out of 22 were validated. Our review of the literature highlights the wide variability across scales used to identify stressors in the ICU with regard to their level of generality/specificity (scales for all types of professional contexts or scales targeting healthcare contexts or more specifically ICU professionals), their psychometric qualities (five levels of validity/reliability), and the type of stressors covered by scales.

This variability sheds light on the constraints that appear to guide investigators' methodological choices. The advantage of using a generic scale validated internationally is that authors are able to carry out epidemiological studies allowing interprofessional and international comparisons (El Khamali et al., 2018). However, generic scales, primarily used in studies, appear ill adapted to measure the stressors more specific to the professional activity in ICUs. Indeed, the stressors in the categories *Dealing with ethical- and moral-related situations* and *Risk management issues*, represented primarily in specific scales, did not appear or were relatively few within the generic and healthcare scales.

For instance, the scale most commonly used by the authors was the JCQ scale, despite the fact that no factor related to *dealing with ethical- and moral-related situations* or *risk management issues* was covered. Many authors, however, raised the question of the importance of the issues related to risk management and patient safety in relation to high-tech care and the severity of the pathology (Aiken and Patrician, 2000; Adriaenssens et al., 2015). The difficulties associated with ethical dimensions have also been extensively studied in the ICU context in relation to end-of-life situations (Laurent et al., 2017). The SAQ-ICU scale combines interesting criteria to identify stress factors in ICU settings. The scale has been validated and covers factors relative to the *problematic relationships with other professionals* and *lack of organizational resources* categories. However, factors relating to stressors such as *job disadvantages, problematic situations with users and relatives, high job demands*, and *risk management issues* are absent, despite the fact that the latter two factors of stress have been widely reported by ICU professionals (Pastores et al., 2019).

Thus, investigators who chose to use a scale reflecting these more specific stressors of ICUs were forced to develop their own tools whose psychometric qualities were yet to be tried and tested (tools based on literature reviews, interviews with professionals, or items from different scales). Beyond the measurement level, these observations highlight the crucial limitation of the uncritical use of general theories of occupational stress (e.g., the job strain model of Karasek, the effort–reward imbalance model of Siegrist) to analyze job stressors in ICUs. Indeed, as advocated by a number of other scholars (Borteyrou et al., 2014), these theories/models of stress must be contextualized to enhance their ecological validity as they only account for generic stressors in professional settings and not the specific ones.

Our systematic review shows that a number of factors are absent from all the scales, for instance, diagnostic/admission decisions, the training and supervision of students, the lack of space related to rooms for break or family discussions (Blanch et al., 2016; Trevick et al., 2016; Pastores et al., 2019). We noted that the healthcare and ICU scales targeted either all healthcare professionals or nurses and that there was no scale developed specifically for physicians.

It is worth nothing that some scales allow the assessment of the effects on outcomes (e.g., job burnout) of the interaction between some types of stressors. This is the case of the JCQ and its variants which can estimate whether resources like job control (decision latitude) and social support (from colleagues and supervisors) moderate the effect of job demands. This is also the case of the ERI scale, which is based on the postulate that the imbalance (interaction) between the rewards obtained in exchange of job efforts is critical to explain outcomes, more than their isolated main effects. However, accounting for these interaction effects generally did not increase the explained variance of outcomes in empirical studies (e.g., Brough and Biggs, 2015; Gorgievski et al., 2019).

This study has a number of limitations. Several studies, and therefore stress scales, may not have been considered in our review if the objective of identifying ICU stressors was not clearly reflected in the article title, keywords, or abstract. In addition, we have focused mainly on studies published in English. Thus, scales developed at a more local level may have been excluded from our review, and a more extensive search seems warranted to cover more the important issue of international, intercultural, and health-system comparisons in terms of stressor identification and measurement.

## Conclusion

Our review of the literature clearly raises the question of the relevance of the scales used in studies measuring stressors in intensive care settings. Indeed, no available tool meets both the criteria of metrological validity and of ecological validity (i.e., covering all relevant stressors in ICUs, particularly those that are the most specific to them). Thus, researchers and practicians currently face a methodological dilemma, as they are forced to make a choice between the two or to make some unfortunate “bricolage,” such as *ad hoc* elaboration, removal, or modification of items or the combination of different scales.

There is an urgent need to propose a validated tool capable of taking into account the whole professional reality of ICU settings. This tool would make it possible to compare the respective impacts of generic vs. specific stressors in the etiology of outcomes (e.g., burnout, job satisfaction, turnover intentions). A better identification of stress factors should make it possible to define a more appropriate care policy in particular to prevent burnout and its associated effects such as depression, suicidal tendencies, addictive behaviors, and physical impairment (e.g., Lheureux et al., 2016; Vandevala et al., 2017). Finally, the more comprehensive and thus more able to approximate the reality of the activities undertaken by professionals the tool is, the easier it will be to use the factors identified to implement effective training needs and target the necessary prevention and support measures.

## Data Availability Statement

All datasets generated for this study are included in the article/Supplementary Material.

## Author Contributions

AL: abstracts screened for eligibility, screened full text sectioned, examination in detail of each scale, examination of typology, and writing of the article. FL: abstracts screened for eligibility, examination in detail of each scale, and writing of the article. MG: initial search and examination in detail of each scale. MM: screened full text sectioned and proofreading. MB: screened full text sectioned and proofreading. AP: screened full text sectioned and proofreading. GB: examination of typology and proofreading. GC: examination of typology and proofreading.

### Conflict of Interest

The authors declare that the research was conducted in the absence of any commercial or financial relationships that could be construed as a potential conflict of interest.

## Footnotes

^1^The formula was: (*N* scales of a given type that measure the stressor/Overall *N* scales that measure the stressor)/(*N* all settings scales that measure the stressor/17) + (*N* healthcare settings scales that measure the stressor/20) + (*N* ICU settings scales that measure the stressor/22). For instance, there were nine scales that measured the “taxing work environment (noisy, hectic, crowded, heated, etc.)” stressor type, including three all settings scales, three healthcare settings scales, and three ICU settings scales. Without the correction, this stressor appeared to be measured to the same extent by the three types of scales (3/9). However, this conclusion is incorrect because the ratio 3/17 (17.65%) for all settings scales is higher than the ratio of 3/20 (15%) for healthcare settings scales and higher than the ratio of 3/22 (13.63%) for ICU settings scales. After the correction which took into account the unequal number of scale types (17 vs. 20 vs. 22), it emerged that this stressor was measured slightly more frequently by all settings scales [(3/17)/((3/17) + (3/20) + (3/22)) = 38%] than by healthcare settings scales [(3/20)/((3/17) + (3/20) + (3/22)) = 35%] and ICU settings scales [(3/22)/((3/17) + (3/20) + (3/22)) = 29%].
