# Supplementary material for: Scales Used to Measure Job Stressors in Intensive Care Units: Are They Relevant and Reliable? A Systematic Review
Source: Front Psychol. 2020 Mar 12;11:245. doi: 10.3389/fpsyg.2020.00245 (PMC7080865; doi:10.3389/fpsyg.2020.00245)
Supplement: Supplementary file 1 [file Table_1.DOCX]

**The 102 articles included in the review**

1. Andolhe R, Barbosa RL, Oliveira EM de, Costa ALS, Padilha KG. Stress, coping and burnout among Intensive Care Unit nursing staff: associated factors. *Rev da Esc Enferm da USP*. 2015;49(spe):58-64. doi:10.1590/S0080-623420150000700009
2. Amin AA, Vankar JR, Nimbalkar SM, Phatak AG. Perceived Stress and Professional Quality of Life in Neonatal Intensive Care Unit Nurses in Gujarat, India. *Indian J Pediatr*. 2015;82(11):1001-1005. doi:10.1007/s12098-015-1794-3
3. Andrade-Nascimento M, Barros DS, Nascimento Sobrinho CL. Professional burnout syndrome among intensive care physicians in salvador, Brazil. *Eur Psychiatry*. 2013;28, Supple:1. doi:http://dx.doi.org/10.1016/S0924-9338(13)76893-7
4. Arikan F, Köksal CD, Gökçe Ç. Work-related stress, burnout, and job satisfaction of dialysis nurses in association with perceived relations with professional contacts. *Dial Transplant*. 2007;36(4):182-191. doi:10.1002/dat.20119
5. Azoulay E, Timsit J-F, Sprung CL, et al. Prevalence and factors of intensive care unit conflicts: the conflicus study. *Am J Respir Crit Care Med*. 2009;180(9):853-860. doi:10.1164/rccm.200810-1614OC
6. Bakker AB, Le Blanc PM, Schaufeli WB. Burnout contagion among intensive care nurses. *J Adv Nurs*. 2005;51(3):276-287. doi:10.1111/j.1365-2648.2005.03494.x
7. Bellagamba G, Gionta G, Senergue J, Bèque C, Lehucher-Michel M-P. Organizational factors impacting job strain and mental quality of life in emergency and critical care units. *Int J Occup Med Environ Health*. 2015;28(2):357-367.
8. Bratt MM, Broome M, Kelber S, Lostocco L. Influence of stress and nursing leadership on job satisfaction of pediatric intensive care unit nurses. *Am J Crit Care*. 2000;9(5):307-317.
9. Burgess L, Irvine F, Wallymahmed A. Personality, stress and coping in intensive care nurses: a descriptive exploratory study. *Nurs Crit Care*. 2010;15(3):129-140. doi:10.1111/j.1478-5153.2009.00384.x
10. Casado Á, Castellanos A, López-Fernández ME, Ruíz R, García Aroca C, Noriega F. Relationship between oxidative and occupational stress and aging in nurses of an intensive care unit. *Age (Omaha)*. 2008;30(4):229-236. doi:10.1007/s11357-008-9052-5
11. Cavaliere TA, Daly B, Dowling D, Montgomery K. Moral distress in neonatal intensive care unit RNs. *Adv Neonatal Care*. 2010;10(3):145-156. doi:10.1097/ANC.0b013e3181dd6c48
12. Cho E, Lee H, Choi M, Park SH, Yoo IY, Aiken LH. Factors associated with needlestick and sharp injuries among hospital nurses: A cross-sectional questionnaire survey. *Int J Nurs Stud*. 2013;50(8):1025-1032. doi:http://dx.doi.org/10.1016/j.ijnurstu.2012.07.009
13. Cho S-H, Park M, Jeon SH, Chang HE, Hong H-J. Average hospital length of stay, nurses’ work demands, and their health and job outcomes. *J Nurs Scholarsh an Off Publ Sigma Theta Tau Int Honor Soc Nurs*. 2014;46(3):199-206. doi:10.1111/jnu.12066
14. Coomber S, Todd C, Park G, Baxter P, Firth-Cozens J, Shore S. Stress in UK intensive care unit doctors. *Br J Anaesth*. 2002;89(6):873-881.
15. Davidson JE, Agan DL, Chakedis S. Exploring Distress Caused by Blame for a Negative Patient Outcome. *J Nurs Adm*. 2016;46(1):18-24. doi:10.1097/NNA.0000000000000288
16. de Boer JC, van Rosmalen J, Bakker AB, van Dijk M. Appropriateness of care and moral distress among neonatal intensive care unit staff: repeated measurements. *Nurs Crit Care*. 2016;21(3):e19-27. doi:10.1111/nicc.12206
17. DePew CL, Gordon M, Yoder LH, Goodwin CW. The relationship of burnout, stress, and hardiness in nurses in a military medical center: a replicated descriptive study. *J Burn Care Rehabil*. 1999;20(6):515-22; discussion 514.
18. de Rijk AE, Le Blanc PM, Schaufeli WB, De Jonge J. Active coping and need for control as moderators of the job demand-control model: Effects on burnout. *J Occup Organ Psychol*. 1998;71(1):1-18.
19. De Villers MJ, DeVon HA. Moral distress and avoidance behavior in nurses working in critical care and noncritical care units. *Nurs Ethics*. 2013;20(5):589-603. doi:10.1177/0969733012452882
20. Elpern EH, Covert B, Kleinpell R. Moral distress of staff nurses in a medical intensive care unit. *Am J Crit Care*. 2005;14(6):523-530. http://www.ncbi.nlm.nih.gov/pubmed/16249589. Accessed April 14, 2017.
21. Embriaco N, Azoulay E, Barrau K, et al. High level of burnout in intensivists: prevalence and associated factors. *Am J Respir Crit Care Med*. 2007;175(7):686-692. doi:10.1164/rccm.200608-1184OC
22. Embriaco N, Hraiech S, Azoulay E, et al. Symptoms of depression in ICU physicians. *Ann Intensive Care*. 2012;2(1):34. doi:10.1186/2110-5820-2-34
23. Fujita S, Ito S, Seto K, Kitazawa T, Matsumoto K, Hasegawa T. Risk factors of workplace violence at hospitals in Japan. *J Hosp Med*. 2012;7(2):79-84. doi:10.1002/jhm.976
24. Ganz FD, Levy H, Khalaila R, et al. Bullying and its prevention among intensive care nurses. *J Nurs Scholarsh*. 2015;47(6):505-511. doi:10.1111/jnu.12167
25. García-Izquierdo M, Ríos-Rísquez MI, Schaufeli WB, Al. E. The relationship between psychosocial job stress and burnout in emergency departments: an exploratory study. *Nurs Outlook*. 2005;60(5):322-329. doi:10.1016/j.outlook.2012.02.002
26. Gauthier T, Meyer RML, Grefe D, Gold JI. An On-the-Job Mindfulness-based Intervention For Pediatric ICU Nurses: A Pilot. *J Pediatr Nurs*. 2015;30(2):402-409. doi:https://doi.org/10.1016/j.pedn.2014.10.005
27. Gibbons C, Geller S, Glatz E. Biomedical equipment in the neonatal intensive care unit: is it a stressor? *J Perinat Neonatal Nurs*. 1998;12(3):67-73.
28. Goetz K, Beutel S, Mueller G, Trierweiler‐Hauke B, Mahler C. Work‐related behaviour and experience patterns of nurses. *Int Nurs Rev*. 2012;59(1):88-93. doi:10.1111/j.1466-7657.2011.00921.x
29. Goodfellow A, Varnam R, Rees D, Shelly MR. Staff stress on the intensive care unit: A comparison of doctors and nurses. *Anaesthesia*. 1997;52(11):1037-1041. doi:10.1111/j.1365-2044.1997.213-az0348.x
30. Gosselin E, Bourgault P, Lavoie S. Association between job strain, mental health and empathy among intensive care nurses. *Nurs Crit Care*. 2016;21(3):137-145. doi:10.1111/nicc.12064
31. Grzeskowiak M, Bartkowska-Sniatkowska A, Rosada-Kurasinska J, Kielbasiewicz-Drozdowska I, Janicki PK. Stress assessment by anaesthesiologists and nurses working in paediatric intensive care units. *East J Med*. 2012;17(2):59-66. http://edergi.yyu.edu.tr/article/view/1049000282. Accessed April 14, 2017.
32. Hamdan M. Measuring safety culture in Palestinian neonatal intensive care units using the Safety Attitudes Questionnaire. *J Crit Care*. 2013;28(5):886.e7-886.e14. doi:http://dx.doi.org/10.1016/j.jcrc.2013.06.002
33. Hamric AB, Blackhall LJ. Nurse-physician perspectives on the care of dying patients in intensive care units: collaboration, moral distress, and ethical climate. *Crit Care Med*. 2007;35(2):422-429. doi:10.1097/01.CCM.0000254722.50608.2D
34. Hansen L, Goodell TT, DeHaven J, Smith M. Nurses’ perceptions of end-of-life care after multiple interventions for improvement. *Am J Crit Care*. 2009;18(3):263-272. doi:10.4037/ajcc2009727
35. Hays MA, All AC, Mannahan C, Cuaderes E, Wallace D. Reported stressors and ways of coping utilized by intensive care unit nurses. *Dimens Crit Care Nurs*. 2006;25(4):185-193.
36. Hooper C, Craig J, Janvrin DR, Wetsel MA, Reimels E. Compassion Satisfaction, Burnout, and Compassion Fatigue Among Emergency Nurses Compared With Nurses in Other Selected Inpatient Specialties. *J Emerg Nurs*. 2010;36(5):420-427. doi:http://dx.doi.org/10.1016/j.jen.2009.11.027
37. Huang DT, Clermont G, Sexton JB, et al. Perceptions of safety culture vary across the intensive care units of a single institution. *Crit Care Med*. 2007;35(1):165-176. doi:10.1097/01.CCM.0000251505.76026.CF
38. Janda R, Jandová E. Symptoms of posttraumatic stress disorder, anxiety and depression among Czech critical care and general surgical and medical ward nurses. *J Res Nurs*. 2015;20(4):298-309. doi:10.1177/1744987115584211
39. Jasper S, Stephan M, Al-Khalaf H, Rennekampff H-O, Vogt PM, Mirastschijski U. Too little appreciation for great expenditure? Workload and resources in ICUs. *Int Arch Occup Environ Health*. 2012;85(7):753-761. doi:10.1007/s00420-011-0721-9
40. Johnson S, Osborn DPJ, Araya R, et al. Morale in the English mental health workforce: questionnaire survey. *Br J Psychiatry*. 2012;201(3):239-246. doi:10.1192/bjp.bp.111.098970
41. Jones G, Hocine M, Salomon J, Dab W, Temime L. Demographic and occupational predictors of stress and fatigue in French intensive-care registered nurses and nurses’ aides: A cross-sectional study. *Int J Nurs Stud*. 2015;52(1):250-259. doi:http://dx.doi.org/10.1016/j.ijnurstu.2014.07.015
42. Karanikola MNK, Albarran JW, Drigo E, et al. Moral distress, autonomy and nurse-physician collaboration among intensive care unit nurses in Italy. *J Nurs Manag*. 2014;22(4):472-484. doi:10.1111/jonm.12046
43. Kawano Y. Association of job-related stress factors with psychological and somatic symptoms among Japanese hospital nurses: effect of departmental environment in acute care hospitals. *J Occup Health*. 2008;50(1):79-85.
44. Kincey J, Lomax M, Shelly M, et al. Sources of Occupational Pressure in Intensive Care and Potential Remedial Interventions. *J Intensive Care Soc*. 2010;11(2):104-108. doi:10.1177/175114371001100206
45. Kinoshita S, Miyashita M. Development of a scale for “difficulties felt by ICU nurses providing end-of-life care” (DFINE): A survey study. *Intensive Crit Care Nurs*. 2011;27(4):202-210. doi:http://dx.doi.org/10.1016/j.iccn.2011.04.006
46. Kinzl JF, Traweger C, Trefalt E, Riccabona U, Lederer W. Work stress and gender-dependent coping strategies in anesthesiologists at a university hospital. *J Clin Anesth*. 2007;19(5):334-338. doi:http://dx.doi.org/10.1016/j.jclinane.2006.08.014
47. Kirkcaldy BD, Martin T. Job stress and satisfaction among nurses: Individual differences. *Stress Med*. 2000;16(2):77-89. doi:10.1002/(SICI)1099-1700(200003)16:2<77::AID-SMI835>3.0.CO;2-Z
48. Klopper HC, Coetzee SK, Pretorius R, Bester P. Practice environment, job satisfaction and burnout of critical care nurses in South Africa. *J Nurs Manag*. 2012;20(5):685-695. doi:10.1111/j.1365-2834.2011.01350.x
49. Le Blanc PM, de Jonge J, de Rijk AE, Schaufeli WB. Well-being of intensive care nurses (WEBIC): a job analytic approach. *J Adv Nurs*. 2001;36(3):460-470.
50. Lee KJ, Forbes ML, Lukasiewicz GJ, et al. Promoting Staff Resilience in the Pediatric Intensive Care Unit. *Am J Crit Care*. 2015;24(5):422-430. doi:10.4037/ajcc2015720
51. Lee SJ, Lee JH, Gillen M, Krause N. Job stress and work-related musculoskeletal symptoms among intensive care unit nurses: A comparison between job demand-control and effort-reward imbalance models. *Am J Ind Med*. 2014;57(2):214-221. doi:10.1002/ajim.22274
52. Leiter MP, Spence Laschinger HK. Relationships of work and practice environment to professional burnout: testing a causal model. *Nurs Res*. 2006;55(2):137-146. http://www.ncbi.nlm.nih.gov/pubmed/16601626. Accessed May 31, 2017.
53. Li J, Lambert VA. Job satisfaction among intensive care nurses from the People’s Republic of China. *Int Nurs Rev*. 2008;55(1):34-39. doi:10.1111/j.1466-7657.2007.00573.x
54. Li J, Lambert VA. Workplace stressors, coping, demographics and job satisfaction in Chinese intensive care nurses. *Nurs Crit Care*. 2008;13(1):12-24. doi:10.1111/j.1478-5153.2007.00252.x
55. Lin T-C, Lin H-S, Cheng S-F, Wu L-M, Ou-Yang M-C. Work stress, occupational burnout and depression levels: a clinical study of paediatric intensive care unit nurses in Taiwan. *J Clin Nurs*. 2016;25(7-8):1120-1130. doi:10.1111/jocn.13119
56. Liu K, You L-M, Chen S-X, et al. The relationship between hospital work environment and nurse outcomes in Guangdong, China: a nurse questionnaire survey. *J Clin Nurs*. 2012;21(9-10):1476-1485. doi:10.1111/j.1365-2702.2011.03991.x
57. Lusignani M, Gianni ML, Re LG, Buffon ML. Moral distress among nurses in medical, surgical and intensive-care units. *J Nurs Manag*. October 2016. doi:10.1111/jonm.12431
58. Malaquin S, Mahjoub Y, Musi A, et al. Burnout syndrome in critical care team members: A monocentric cross sectional survey. *Anaesth Crit Care Pain Med*. doi:http://dx.doi.org/10.1016/j.accpm.2016.06.011
59. Martins Pereira S, Teixeira CM, Carvalho AS, Hernandez-Marrero P. Compared to Palliative Care, Working in Intensive Care More than Doubles the Chances of Burnout: Results from a Nationwide Comparative Study. *PLoS One*. 2016;11(9):e0162340. doi:10.1371/journal.pone.0162340
60. Mason VM, Leslie G, Clark K, et al. Compassion fatigue, moral distress, and work engagement in surgical intensive care unit trauma nurses: a pilot study. *Dimens Crit Care Nurs*. 2014;33(4):215-225. doi:10.1097/DCC.0000000000000056
61. McCarthy VJC, Power S, Greiner BA. Perceived occupational stress in nurses working in Ireland. *Occup Med (Chic Ill)*. 2010;60(8):604-610. doi:10.1093/occmed/kqq148
62. Meltzer LS, Huckabay LM. Critical care nurses’ perceptions of futile care and its effect on burnout. *Am J Crit Care*. 2004;13(3):202-208. http://www.ncbi.nlm.nih.gov/pubmed/15149054. Accessed April 14, 2017.
63. Mohammadi M, Mazloumi A, Kazemi Z, Zeraati H. Evaluation of Mental Workload among ICU Ward’s Nurses. *Heal Promot Perspect*. 2015;5(4):280-287. doi:10.15171/hpp.2015.033
64. Morelius E, Gustafsson PA, Ekberg K, Nelson N. Neonatal intensive care and child psychiatry inpatient care: do different working conditions influence stress levels? *Nurs Res Pract*. 2013;2013:761213. doi:10.1155/2013/761213
65. Mrayyan MT. Job stressors and social support behaviors: comparing intensive care units to wards in Jordan. *Contemp Nurse*. 2009;31(2):163-175.
66. Myhren H, Ekeberg O, Stokland O. Job Satisfaction and Burnout among Intensive Care Unit Nurses and Physicians. *Crit Care Res Pract*. 2013;2013:786176. doi:10.1155/2013/786176
67. O’Brien-Pallas L, Murphy GT, Shamian J, Li X, Hayes LJ. Impact and determinants of nurse turnover: a pan-Canadian study. *J Nurs Manag*. 2010;18(8):1073-1086. doi:10.1111/j.1365-2834.2010.01167.x
68. Papathanassoglou EDE, Karanikola MNK, Kalafati M, Giannakopoulou M, Lemonidou C, Albarran JW. Professional autonomy, collaboration with physicians, and moral distress among European intensive care nurses. *Am J Crit Care*. 2012;21(2):e41-52. doi:10.4037/ajcc2012205
69. Park M, Cho S-H, Hong H-J. Prevalence and perpetrators of workplace violence by nursing unit and the relationship between violence and the perceived work environment. *J Nurs Scholarsh an Off Publ Sigma Theta Tau Int Honor Soc Nurs*. 2015;47(1):87-95. doi:10.1111/jnu.12112
70. Pereira Rodrigues VMC, de Sousa Ferreira AS. Stressors in nurses working in Intensive Care Units. *Rev Lat Am Enfermagem*. 2011;19(4):1025-1032. doi:10.1590/S0104-11692011000400023
71. Piers RD, Azoulay E, Ricou B, et al. Perceptions of appropriateness of care among European and Israeli intensive care unit nurses and physicians. *JAMA*. 2011;306(24):2694-2703. doi:10.1001/jama.2011.1888
72. Poley MJ, van der Starre C, van den Bos A, van Dijk M, Tibboel D. Patient safety culture in a Dutch pediatric surgical intensive care unit: An evaluation using the Safety Attitudes Questionnaire. *Pediatr Crit Care Med*. 2011;12(6):e310-e316. doi:https://doi.org/10.1097/PCC.0b013e318220afca
73. Poncet MC, Toullic P, Papazian L, et al. Burnout syndrome in critical care nursing staff. *Am J Respir Crit Care Med*. 2007;175(7):698-704. doi:10.1164/rccm.200606-806OC
74. Profit J, Sharek PJ, Amspoker AB, et al. Burnout in the NICU setting and its relation to safety culture. *BMJ Qual Saf*. 2014;23(10):806-813. doi:10.1136/bmjqs-2014-002831
75. Qin Z, Zhong X, Ma J, Lin H. Stressors affecting nurses in China. *Contemp Nurse*. 2016;52(4):447-453. doi:10.1080/10376178.2016.1221321
76. Robert R, Le Gouge A, Kentish-Barnes N, et al. Terminal weaning or immediate extubation for withdrawing mechanical ventilation in critically ill patients (the ARREVE observational study). *Intensive Care Med*. 2017;43(12):1793-1807. doi:10.1007/s00134-017-4891-0
77. Rochefort CM, Clarke SP. Nurses’ work environments, care rationing, job outcomes, and quality of care on neonatal units. *J Adv Nurs*. 2010;66(10):2213-2224. doi:10.1111/j.1365-2648.2010.05376.x
78. Sagie A, Krausz M. What aspects of the job have most effect on nurses? *Hum Resour Manag J*. 2003;13(1):46-62. doi:10.1111/j.1748-8583.2003.tb00083.x
79. Saini R, Kaur S, Das K. Assessment of stress and burnout among intensive care nurses at a tertiary care hospital. *J Ment Heal Hum Behav*. 2011;16(1):43-48.
80. Schwarzkopf D, Westermann I, Skupin H, et al. A novel questionnaire to measure staff perception of end-of-life decision making in the intensive care unit—Development and psychometric testing. *J Crit Care*. 2015;30(1):187-195. doi:http://dx.doi.org/10.1016/j.jcrc.2014.09.015
81. Shehabi Y, Dobb G, Jenkins I, Pascoe R, Edwards N, Butt W. Burnout syndrome among Australian intensivists: a survey. *Crit Care Resusc*. 2008;10(4):312-315.
82. Shoorideh FA, Ashktorab T, Yaghmaei F, Alavi Majd H. Relationship between ICU nurses’ moral distress with burnout and anticipated turnover. *Nurs Ethics*. 2015;22(1):64-76. doi:10.1177/0969733014534874
83. Teixeira C, Ribeiro O, Fonseca AM, Carvalho AS. Ethical decision making in intensive care units: a burnout risk factor? Results from a multicentre study conducted with physicians and nurses. *J Med Ethics*. 2014;40(2):97-103. doi:10.1136/medethics-2012-100619
84. Teixeira C, Ribeiro O, Fonseca AM, Carvalho AS. Burnout in intensive care units - a consideration of the possible prevalence and frequency of new risk factors: a descriptive correlational multicentre study. *BMC Anesthesiol*. 2013;13(1):38. doi:10.1186/1471-2253-13-38
85. Tolomiczenko GS, Kahan M, Ricci M, et al. SARS: coping with the impact at a community hospital. *J Adv Nurs*. 2005;50(1):101-110. doi:10.1111/j.1365-2648.2005.03366.x
86. Trotochaud K, Coleman JR, Krawiecki N, McCracken C. Moral Distress in Pediatric Healthcare Providers. *J Pediatr Nurs*. 2015;30(6):908-914. doi:10.1016/j.pedn.2015.03.001
87. Trousselard M, Dutheil F, Naughton G, et al. Stress among nurses working in emergency, anesthesiology and intensive care units depends on qualification: a Job Demand-Control survey. *Int Arch Occup Environ Health*. 2016;89(2):221-229. doi:10.1007/s00420-015-1065-7
88. Tummers GER, van Merode GG, Landeweerd JA. The diversity of work: differences, similarities and relationships concerning characteristics of the organisation, the work and psychological work reactions in intensive care and non-intensive care nursing. *Int J Nurs Stud*. 2002;39(8):841-855. doi:http://dx.doi.org/10.1016/S0020-7489(02)00020-2
89. Tummers GER, van Merode GG, Landeweerd JA. Organizational Characteristics as Predictors of Nurses’ Psychological Work Reactions. *Organ Stud*. 2006;27(4):559-584. doi:10.1177/0170840605059455
90. van Dam K, Meewis M, van der Heijden BIJM. Securing intensive care: towards a better understanding of intensive care nurses’ perceived work pressure and turnover intention. *J Adv Nurs*. 2013;69(1):31-40. doi:10.1111/j.1365-2648.2012.05981.x
91. Vandevala T, Pavey L, Chelidoni O, Chang N-F, Creagh-Brown B, Cox A. Psychological rumination and recovery from work in intensive care professionals: associations with stress, burnout, depression and health. *J Intensive Care*. 2017;5. doi:10.1186/s40560-017-0209-0
92. Verdon M, Merlani P, Perneger T, Ricou B. Burnout in a surgical ICU team. *Intensive Care Med*. 2008;34(1):152-156. doi:10.1007/s00134-007-0907-5
93. Verhaeghe R, Vlerick P, De Backer G, Van Maele G, Gemmel P. Recurrent changes in the work environment, job resources and distress among nurses: A comparative cross-sectional survey. *Int J Nurs Stud*. 2008;45(3):382-392. doi:http://dx.doi.org/10.1016/j.ijnurstu.2006.10.003
94. Vessey JA, DeMarco RF, Gaffney DA, Budin WC. Bullying of Staff Registered Nurses in the Workplace: A Preliminary Study for Developing Personal and Organizational Strategies for the Transformation of Hostile to Healthy Workplace Environments. *J Prof Nurs*. 2009;25(5):299-306. doi:https://doi.org/10.1016/j.profnurs.2009.01.022
95. Weigl M, Schneider A, Hoffmann F, Angerer P. Work stress, burnout, and perceived quality of care: a cross-sectional study among hospital pediatricians. *Eur J Pediatr*. 2015;174(9):1237-1246. doi:10.1007/s00431-015-2529-1
96. Welp A, Meier LL, Manser T. The interplay between teamwork, clinicians’ emotional exhaustion, and clinician-rated patient safety: a longitudinal study. *Crit Care*. 2016;20(1):110. doi:10.1186/s13054-016-1282-9
97. Welp A, Meier LL, Manser T. Emotional exhaustion and workload predict clinician-rated and objective patient safety. *Front Psychol*. 2015;5.
98. Whitehead PB, Herbertson RK, Hamric AB, Epstein EG, Fisher JM. Moral distress among healthcare professionals: report of an institution-wide survey. *J Nurs Scholarsh an Off Publ Sigma Theta Tau Int Honor Soc Nurs*. 2015;47(2):117-125. doi:10.1111/jnu.12115
99. Wilson MA, Goettemoeller DM, Bevan NA, Mccord JM. Moral distress: Levels, coping and preferred interventions in critical care and transitional care nurses. *J Clin Nurs*. 2013;22(9-10):1455-1466. doi:10.1111/jocn.12128
100. Wykes T, Whittington R. Prevalence and predictors of early traumatic stress reactions in assaulted psychiatric nurses. *J Forensic Psychiatry*. 1998;9(3):643-658. doi:10.1080/09585189808405379
101. Yamase H. Development of a comprehensive scoring system to measure multifaceted nursing workloads in ICU. *Nurs Health Sci*. 2003;5(4):299-308. doi:10.1046/j.1442-2018.2003.00165.x
102. Zenere A, Zanolin ME, Negri R, Moretti F, Grassi M, Tardivo S. Assessing safety culture in NICU: Psychometric properties of the Italian version of Safety Attitude Questionnaire and result implications. *J Eval Clin Pract*. 2016;22(2):275-282. doi:10.1111/jep.12472
